# Ten-Year Clinical Outcomes and Valve Durability After Transcatheter Aortic Valve Implantation With Balloon-Expandable Valves

**DOI:** 10.1016/j.jacasi.2026.02.003

**Published:** 2026-03-14

**Authors:** Akihiro Ikuta, Yasushi Fuku, Satoki Oka, Shunsuke Matsushita, Futoshi Yamanaka, Shinichi Shirai, Norio Tada, Toru Naganuma, Masahiro Yamawaki, Masahiko Asami, Hirofumi Hioki, Toshiaki Otsuka, Yusuke Watanabe, Masanori Yamamoto, Kentaro Hayashida, OCEAN–TAVI Investigators

**Affiliations:** a Department of Cardiovascular Medicine, Kurashiki Central Hospital, Kurashiki, Japan; b Department of Cardiology, Shonan Kamakura General Hospital, Kamakura, Japan; c Department of Cardiology, Kokura Memorial Hospital, Kitakyushu, Japan; d Department of Cardiology, Sendai Kosei Hospital, Sendai, Japan; e Department of Cardiology, New Tokyo Hospital, Matsudo, Japan; f Department of Cardiology, Tokai University School of Medicine, Isehara, Japan; g Division of Cardiology, Mitsui Memorial Hospital, Tokyo, Japan; h Department of Cardiology, IMS Tokyo Katsushika General Hospital, Tokyo, Japan; i Department of Hygiene and Public Health, Nippon Medical School, Tokyo, Japan; j Department of Cardiology, Teikyo University School of Medicine, Tokyo, Japan; k Department of Cardiology, Toyohashi Heart Center, Toyohashi, Japan; l Department of Cardiology, Nagoya Heart Center, Nagoya, Japan; m Department of Cardiology, Gifu Heart Center, Gifu, Japan; n Department of Cardiology, Keio University School of Medicine, Tokyo, Japan

**Keywords:** balloon expandable valve, bioprosthetic valve failure, transcatheter aortic valve implantation

## Abstract

**Background:**

Transcatheter aortic valve implantation (TAVI) is a well-established treatment for patients with severe aortic stenosis. However, long-term data exceeding 10 years remain scarce.

**Objectives:**

This study aimed to evaluate long-term clinical outcomes and transcatheter heart valve durability in patients who underwent TAVI and completed a 10-year follow-up.

**Methods:**

Using data from the multicenter registry in Japan, we analyzed outcomes of 297 patients who received the SAPIEN XT valve (Edwards Lifesciences) from June 2010 to June 2014. All-cause mortality was the primary endpoint. Valve-related outcomes, including bioprosthetic valve failure, were defined according to the Valve Academic Research Consortium 3 criteria.

**Results:**

The mean age of the participants was 83.8 ± 6.0 years, and the mean Society of Thoracic Surgeons risk score was 7.5 ± 4.0. Freedom from all-cause mortality at 10 years was 13.2%. Severe structural valve deterioration and bioprosthetic valve failure gradually increased up to 10 years, reaching 3.0% and 10.8%, respectively. Reintervention was performed in 4.5% of patients. Valve-related deaths accounted for 1.3% of all deaths. Most deaths were due to non–valve-related causes. Multivariate analysis revealed that high Society of Thoracic Surgeons score, high clinically frailty scale, renal dysfunction, and low serum albumin levels were independent predictors of mortality.

**Conclusions:**

Few older adult high-risk patients survived beyond 10 years following TAVI. In contrast, transcatheter heart valve demonstrated acceptable durability, and long-term outcomes were primarily determined by baseline comorbidities rather than valve dysfunction. These results support the role of TAVI as a feasible therapeutic approach in this population.

In patients with severe aortic stenosis, transcatheter aortic valve implantation (TAVI) has become a well-known therapeutic strategy, with procedural volumes continuing to increase worldwide.^1^ This trend has been mainly attributed to results from large-scale clinical trials demonstrating favorable outcomes of TAVI compared with surgical valve replacement across all risk categories.^2^, ^3^, ^4^ Notably, favorable outcomes in low-risk populations have further accelerated the adoption of TAVI in this cohort. However, long-term outcomes, including valve durability, have become a critical concern. During the early era of TAVI, the procedure was predominantly performed in inoperable or high-risk patients with limited life expectancy, making long-term durability assessment difficult owing to the competing risk of mortality. Consequently, 10-year data remain limited.^5^^,^^6^ In this context, evaluating mortality and valve-related events over a 10-year period in high-risk patients treated with first-generation transcatheter heart valves (THVs) is crucial for informing future expectations of valve performance in broader patient populations. This study aimed to evaluate long-term clinical outcomes and THV durability at 10 years following TAVI using early-generation balloon-expandable valves, leveraging data from a multicenter registry in Japan.

## Methods

### Study design and population

**Figure 1:**
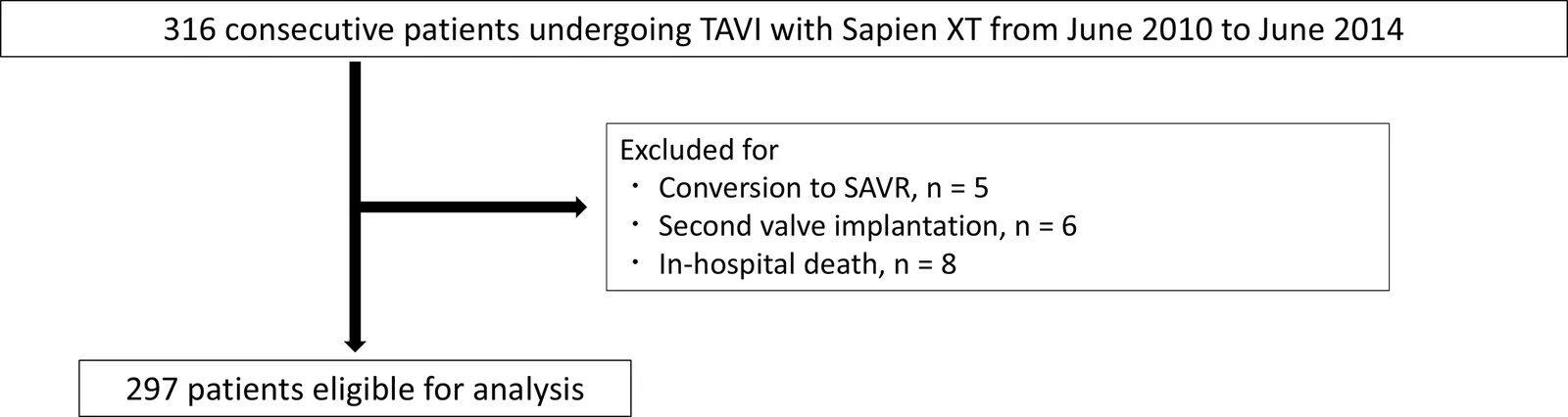
Study Flow Chart Of 316 patients with severe aortic stenosis who underwent transcatheter aortic valve implantation (TAVI) with the SAPIEN XT valve from 2010 to 2014, 19 were excluded based on predefined criteria. Ultimately 297 patients were included. SAVR = surgical aortic valve replacement.

This study was conducted using data from the OCEAN-TAVI (Optimized Catheter Valvular Intervention–Transcatheter Aortic Valve Implantation) registry, an ongoing prospective multicenter registry in Japan. All patients were either inoperable or at high risk for surgical aortic valve replacement (SAVR) by consensus between individual centers and after discussions within the local heart team. The study population comprised 316 patients with severe aortic stenosis who underwent TAVI with the SAPIEN XT valve (Edwards Lifesciences) from June 2010 to June 2014 from 11 institutions in Japan. After excluding patients who underwent conversion to SAVR during hospitalization (n = 5), second valve implantation during hospitalization (n = 6), or in-hospital death (n = 8), 297 patients were included in the final analysis (Figure 1). The study protocol of the OCEAN-TAVI registry was developed in accordance with the Declaration of Helsinki and was approved by the local institutional review boards of the participating centers. Written informed consent was obtained from all patients before undergoing TAVI. This clinical trial was registered with the University Hospital Medical Information Network (UMIN00020423).

### Data collection and clinical endpoints

Data from the OCEAN-TAVI registry, including baseline patient characteristics, laboratory data, echocardiographic data, procedural variables, and clinical outcomes with respect to mortality rehospitalization, as well as other clinical adverse events, were prospectively recorded. Data on the possible occurrence and/or causes of adverse events were obtained from either the medical records of each center or treating hospital team by either interview at a planned hospital visit or by telephone interview and questionnaire. All clinical endpoints, procedural data, complications, postprocedural parameters, and in-hospital events were defined using the Valve Academic Research Consortium 3 criteria.^7^

All-cause mortality was the primary clinical endpoint; other relevant clinical outcomes included cardiovascular mortality, heart failure hospitalization, and stroke. Echocardiographic outcomes were the effective orifice area (EOA) of the THVs and the mean pressure gradient (mPG), the degree of central regurgitation, and paravalvular leak (PVL). Structural valve deterioration (SVD) was differentiated into 3 degrees of severity. Stage 1 denotes morphological valve dysfunction without evidence of hemodynamic changes. Stage 2 indicates moderate hemodynamic valve deterioration, defined as an increased mPG of ≥10.0 mm Hg compared with baseline, leading to an mPG of ≥20.0 mm Hg, along with a decreased EOA of ≥0.3 cm^2^ or ≥25.0%, or an increase of ≥1 degree of intraprosthetic aortic regurgitation (AR), resulting in ≥ moderate AR. Severe structural valve deterioration (SVD), corresponding to stage 3, refers to an increased mPG of ≥20.0 mm Hg resulting in an mPG of ≥30.0 mm Hg, along with a decreased EOA of ≥0.6 cm^2^ or ≥50.0%, or an increase of 2 grades, resulting in severe intraprosthetic AR.

Bioprosthetic valve dysfunction (BVD) encompassed the following 4 components: 1) SVD; 2) non-SVD (PVL and prosthesis–patient mismatch [PPM]); 3) clinical valve thrombosis; and 4) endocarditis following transcatheter aortic valve replacement. Bioprosthetic valve failure (BVF) was defined as one of the following situations: 1) any BVD with new-onset or worsening symptoms, pulmonary hypertension, or irreversible stage 3 hemodynamic valve deterioration; 2) aortic valve reintervention (surgical explant and replacement or transcatheter aortic valve) following BVD diagnosis; or 3) valve-related death (death presumably associated with BVD or sudden unexplained death following BVD diagnosis). The degree of PVL was classified into 3 grades as follows: none/trivial, mild, and greater than or equal to moderate. Postprocedural valve performance was evaluated using the EOA, indexed EOA, peak flow velocity, mPG, and PPM incidence. PPM severity was categorized as no PPM, moderate PPM, or severe PPM based on EOA values and patient body mass index according to the Valve Academic Research Consortium 3 definition. PPM and postoperative hemodynamics were assessed based on echocardiographic examinations performed within 1 week following TAVI in all cases.

### Statistical analysis

Categorical variables were expressed as counts (%) and compared using the chi square test. Continuous variables were presented as means ± SDs and compared using Student’s *t*-test or the Wilcoxon rank-sum test, as appropriate. Continuous variables were compared between survivors and nonsurvivors during follow-up. Data were presented as means ± SDs or medians (Q1-Q3) for continuous variables. The Kaplan–Meier plot was used to describe the cumulative mortality and rate of cardiac death between categories. Furthermore, to comprehensively assess heart failure hospitalization, stroke, BVD, BVF, SVD, and reintervention, we used the Fine–Gray test for analyzing all-cause death as a competitive risk. The proportional subdistribution hazards assumption was evaluated by incorporating time-dependent interaction terms between covariates and follow-up time in the Fine–Gray models. Continuous covariates were modeled as linear terms. Potential nonlinearity of age was assessed using restricted cubic spline functions, which did not show meaningful departures from linearity. Censoring was primarily administrative at the end of follow-up, with minimal loss to follow-up. Therefore, censoring was assumed to be independent of the event of interest. Key covariates were selected a priori based on clinical relevance and prior literature to obtain the final model for individuating independent variables associated with all-cause mortality at 10 years following TAVI. All statistical analyses were performed using JMP version 14.0 (SAS Institute) and R version 4.2.2 (R Foundation for Statistical Computing). A 2-tailed *P* < 0.05 was considered statistically significant.

## Results

### Baseline patient characteristics and procedural outcomes

Baseline patient characteristics and procedural outcomes are summarized in Tables 1 and 2, respectively. The mean age of the participants was 83.8 ± 6.0 years, 28.3% of whom were male, and the mean Society of Thoracic Surgeons (STS) score was 7.5 ± 4.0. The transfemoral approach was performed in 83.2% of the participants, and the 23-mm valve size accounted for 69% of the procedures.

**Table 1:** Baseline Patient Characteristics

	Overall (N = 297)	Alive (n = 93)	Dead (n = 204)	*P* Value
Age, y	83.8 ± 6.0	82.2 ± 6.2	84.5 ± 4.2	<0.01
Age ≥85	159 (53.5)	37 (39.8)	122 (59.8)	<0.01
Men	84 (28.3)	27 (29.0)	57 (27.9)	0.85
Height, cm	149.8 ± 9.0	148.5 ± 8.9	149.6 ± 9.1	0.35
Weight, kg	49.9 ± 9.9	52.0 ± 11.5	49.0 ± 8.9	0.01
BSA, m^2^	1.43 ± 0.16	1.45 ± 0.19	1.41 ± 0.14	0.03
BMI, kg/m^2^	22.3 ± 3.8	22.9 ± 4.0	22.0 ± 3.6	0.048
NYHA functional class				0.02
Ⅰ/Ⅱ	130 (43.9)	50 (54.4)	80 (39.2)	
Ⅲ/Ⅳ	166 (56.1)	42 (45.7)	124 (60.8)	
Clinical frailty scale				0.59
≤3	110 (37.5)	37 (39.8)	73 (36.5)	
≥4	183 (62.5)	56 (60.2)	127 (63.5)	
Society of Thoracic Surgeons risk score, %	7.5 ± 4.0	6.1 ± 3.5	8.2 ± 4.1	<0.01
Hypertension	236 (79.5)	73 (78.5)	163 (79.9)	0.78
Diabetes mellitus	85 (28.6)	30 (32.3)	55 (27.0)	0.35
Dyslipidemia	171 (57.6)	66 (71.0)	105 (51.5)	0.01
Previous myocardial infarction	26 (8.8)	10 (10.8)	16 (7.8)	0.42
Previous cardiac surgery	44 (29.9)	19 (38.8)	25 (25.5)	0.10
Previous ischemic stroke	31 (10.4)	11 (11.8)	20 (9.8)	0.60
Coronary artery disease	126 (42.4)	41 (44.1)	85 (41.7)	0.70
Peripheral artery disease	46 (15.5)	10 (10.8)	36 (17.7)	0.12
Chronic kidney disease	231 (77.8)	65 (69.9)	166 (81.4)	0.03
Chronic obstructive pulmonary disease	33 (11.1)	16 (17.2)	17 (8.3)	0.03
Atrial fibrillation	69 (23.2)	16 (17.2)	53 (26.0)	0.09
Permanent pacemaker	22 (7.4)	6 (6.5)	16 (7.8)	0.67
Laboratory data				
Serum albumin, g/dL	3.8 ± 0.6	4.0 ± 0.6	3.8 ± 0.5	<0.01
Hemoglobin, g/dL	11.1 ± 1.6	11.6 ± 1.5	10.9 ± 1.6	<0.01
Creatinine, mg/dL	1.2 ± 2.2	0.9 ± 0.3	1.3 ± 2.6	0.15
eGFR, mL/min/1.73 m^2^	49.2 ± 18.2	54.9 ± 16.6	46.6 ± 18.4	<0.01
B-type natriuretic peptide, pg/mL	411 ± 493	296 ± 315	465 ± 550	0.01
Echocardiographic parameters				
Left ventricular ejection fraction, %	60.7 ± 11.7	62.0 ± 10.6	60.0 ± 12.2	0.19
Left ventricular end-diastolic diameter, mm	44.5 ± 6.2	44.8 ± 5.7	44.3 ± 6.5	0.48
Left ventricular end-systolic diameter, mm	29.7 ± 6.8	29.6 ± 5.8	29.7 ± 7.2	0.84
Mean aortic gradient, mm Hg	50.4 ± 18.2	49.8 ± 17.1	50.6 ± 18.7	0.74
Aortic valve area, cm^2^	0.62 ± 0.16	0.65 ± 0.16	0.61 ± 0.16	0.11
Aortic regurgitation ≥ mild	198 (66.7)	61 (65.6)	137 (67.2)	0.79
Aortic regurgitation ≥ moderate	42 (14.1)	17 (18.3)	25 (12.3)	0.21
Computed tomography parameters				
Annulus area, mm^2^	389 ± 61	391 ± 57	387 ± 62	0.60
Annulus perimeter, mm	71.5 ± 5.4	71.6 ± 4.8	71.5 ± 5.6	0.79

Values are mean ± SD or n (%), unless otherwise specified.

BMI = body mass index; BSA = body surface area, eGFR = estimated glomerular filtration rate.

**Table 2:** Procedural Results and In-Hospital Outcomes

	Overall (N = 297)	Alive (n = 93)	Dead (n = 204)	*P* Value
Urgent or emergent procedure	3 (1.0)	0 (0)	3 (1.5)	0.13
General anesthesia	293 (98.7)	93 (100)	200 (98.0)	0.08
Transfemoral	247 (83.2)	84 (90.3)	163 (79.9)	0.02
Prosthesis size, mm				0.47
20	2 (0.7)	0 (0)	2 (1.0)	
23	205 (69.0)	65 (69.9)	140 (68.6)	
26	90 (30.3)	28 (30.1)	62 (30.4)	
Balloon predilation	288 (97.0)	93 (100)	195 (95.6)	0.01
Balloon postdilation	84 (28.3)	29 (31.2)	55 (27.0)	0.46
Contrast volume, mL	143 ± 67	159 ± 76	136 ± 62	0.01
Blood transfusion	100 (33.7)	25 (26.9)	75 (36.8)	0.09
Echocardiographic parameters at discharge				
Postprocedural mean aortic gradient, mm Hg	10.8 ± 4.2	11.0 ± 4.0	10.7 ± 4.3	0.57
Effective orifice area, cm^2^	1.67 ± 0.36	1.62 ± 0.39	1.69 ± 0.35	0.64
Aortic regurgitation ≥ mild	105 (35.4)	36 (38.7)	69 (33.8)	0.43
In-hospital outcome				
Life-threatening/disabling bleeding	14 (4.7)	2 (2.2)	12 (5.9)	0.13
Major bleeding	27 (9.1)	6 (6.5)	21 (10.3)	0.01
Minor bleeding	36 (12.1)	8 (8.6)	28 (13.7)	0.20
Major vascular complication	13 (4.4)	3 (3.2)	10 (4.9)	0.76
Minor vascular complication	9 (3.0)	3 (3.2)	6 (2.9)	0.89
Acute kidney injury	26 (8.8)	6 (6.5)	20 (9.8)	0.33
Disabling stroke	2 (0.7)	0 (0)	2 (1.0)	0.85
Coronary obstruction	3 (1.0)	1 (1.1)	2 (1.0)	0.94
New pacemaker implantation	16 (5.8)	4 (4.3)	12 (5.9)	0.57

Values are mean ± SD or n (%), unless otherwise specified.

### Long-term clinical outcomes

**Figure 2:**
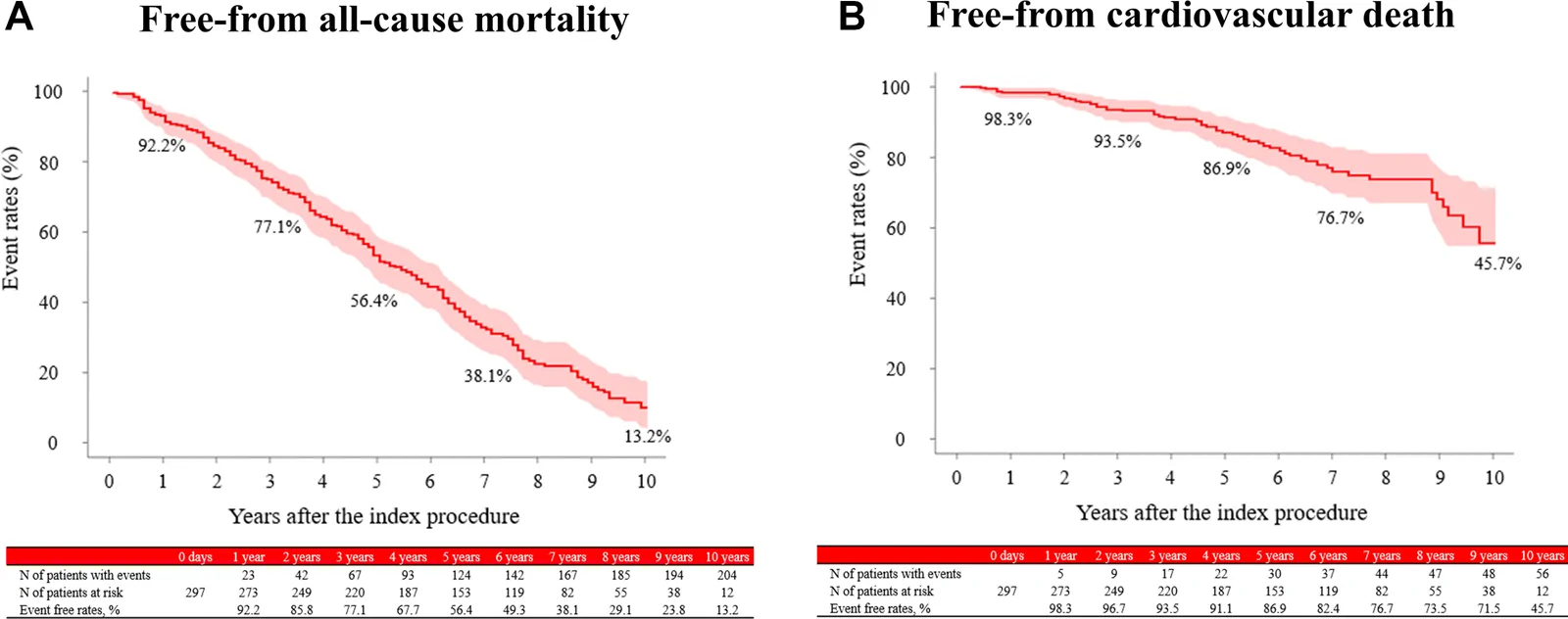
Ten-Year Cumulative Incidence of All-Cause Mortality and Cardiovascular Mortality Cumulative incidence curves showing the incidence of (A) free-from all-cause mortality and (B) free-from cardiovascular mortality.

**Figure 3:**
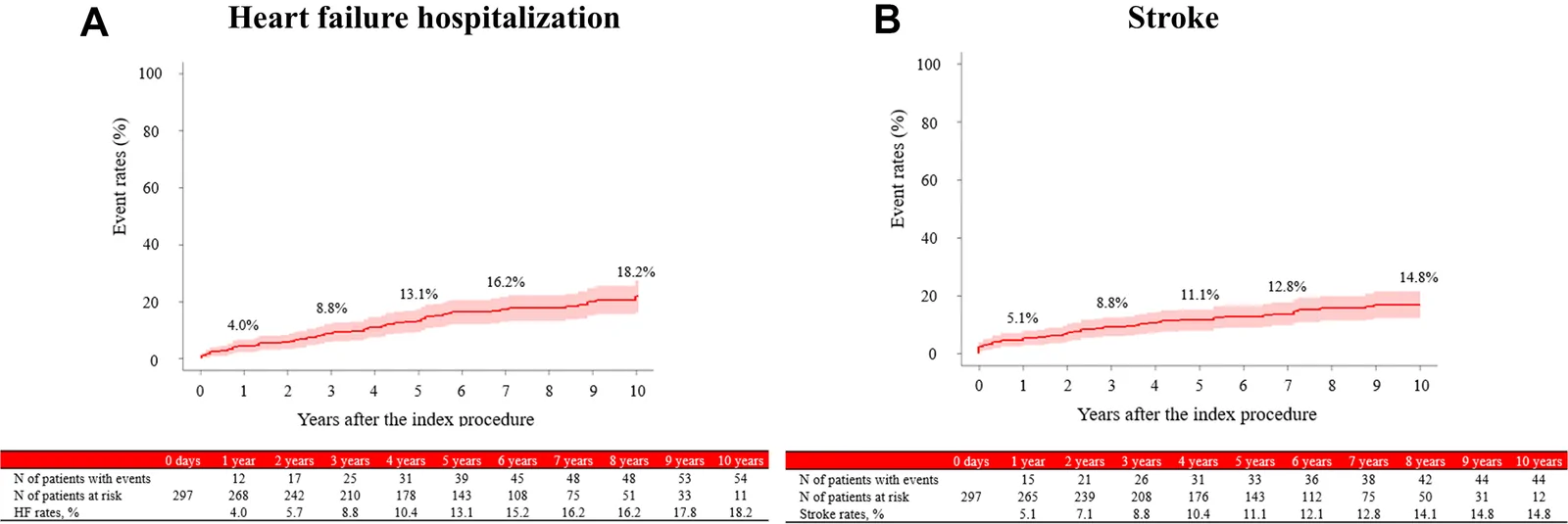
Ten-Year Cumulative Incidence of Heart Failure Hospitalization and Stroke Cumulative incidence curves using the Fine–Gray competing risk model showing the incidence of (A) heart failure hospitalization and (B) stroke over the 10-year follow-up. HF = heart failure.

The median follow-up duration was 5.0 (Q1-Q3: 2.9-7.3) years. The overall survival rates at 1, 5, and 10 years were 92.2%, 56.4%, and 13.2%, respectively. The rates of freedom from cardiovascular mortality at 1, 5, and 10 years were 93.5%, 86.9%, and 45.7%, respectively (Figure 2). To comprehensively investigate heart failure hospitalization and stroke events, a competing risk analysis using the Fine–Gray model was performed, and all-cause death was treated as a competing event. The cumulative incidences of heart failure hospitalization at 1, 5, and 10 years were 4.0%, 13.1%, and 18.2%, respectively. The cumulative incidences of stroke at 1, 5, and 10 years were 5.1%, 11.1%, and 14.8%, respectively (Figure 3).

The univariate and multivariate variables of all-cause mortality are presented in Table 3. Multivariate analysis identified STS score (HR: 1.41; 95% CI: 1.13-1.51; *P* < 0.01), clinical frailty scale (CFS) (HR: 1.26; 95% CI: 1.10-1.44; *P* < 0.01), estimated glomerular filtration rate (eGFR) (HR: 1.01; 95% CI: 1.01-1.02; *P* = 0.01), and serum albumin level (HR: 2.06; 95% CI: 1.53-2.77; *P* < 0.01) as independent predictors of all-cause mortality at 10 years following TAVI.

**Table 3:** Univariate and Multivariate Variables of All-Cause Death at 10 Years Following TAVI

	Univariate Analysis		Multivariate Analysis	
	HR (95% CI)	*P* Value	HR (95% CI)	*P* Value
Age (per 1-y increase)	1.05 (1.03-1.08)	<0.01	1.01 (0.98-1.04)	0.59
Males	0.95 (0.55-1.14)	0.85		
BMI (per 1-kg/m^2^ decrease)	1.06 (1.02-1.10)	0.01	1.04 (0.99-1.09)	0.06
Clinical frailty scale (per 1-point increase)	1.29 (1.14-1.45)	<0.01	1.26 (1.10-1.44)	<0.01
STS risk score (per 1-point increase)	1.21 (1.12-1.38)	<0.01	1.41 (1.13-1.51)	<0.01
NYHA functional class III/IV	1.42 (1.07-1.88)	0.02	1.13 (0.97-1.46)	0.08
Hypertension	1.09 (0.59-1.97)	0.78		
Diabetes mellitus	0.78 (0.46-1.33)	0.35		
Previous myocardial infarction	1.02 (0.61-1.67)	0.95		
Peripheral artery disease	1.22 (0.85-1.75)	0.28		
Atrial fibrillation	1.54 (1.12-2.11)	0.01	1.36 (0.97-1.91)	0.07
Hemoglobin (per 1-g/dL decrease)	1.16 (1.06-1.27)	<0.01	1.04 (0.97-1.06)	0.08
eGFR (per 1-mL/min/1.73 m^2^ decrease)	1.02 (1.01-1.03)	<0.01	1.01 (1.01-1.02)	0.01
Serum albumin (per 1-g/dL decrease)	2.18 (1.66-2.84)	<0.01	2.06 (1.53-2.77)	<0.01
B-type natriuretic peptide (per 500-pg/mL increase)	1.78 (1.22-2.76)	<0.01	1.15 (0.98-1.33)	0.12
Left ventricular ejection fraction (per 1.0% decrease)	1.01 (0.96-1.01)	0.19		
Transfemoral approach	0.74 (0.52-1.04)	0.08	0.81 (0.67-1.12)	0.11

Values are mean ± SD or n (%), unless otherwise specified.

eGFR = estimated glomerular filtration rate; STS = Society of Thoracic Surgeons risk score; TAVI = transcatheter aortic valve implantation; other abbreviation as in Table 1.

### THV function and durability

**Figure 4:**
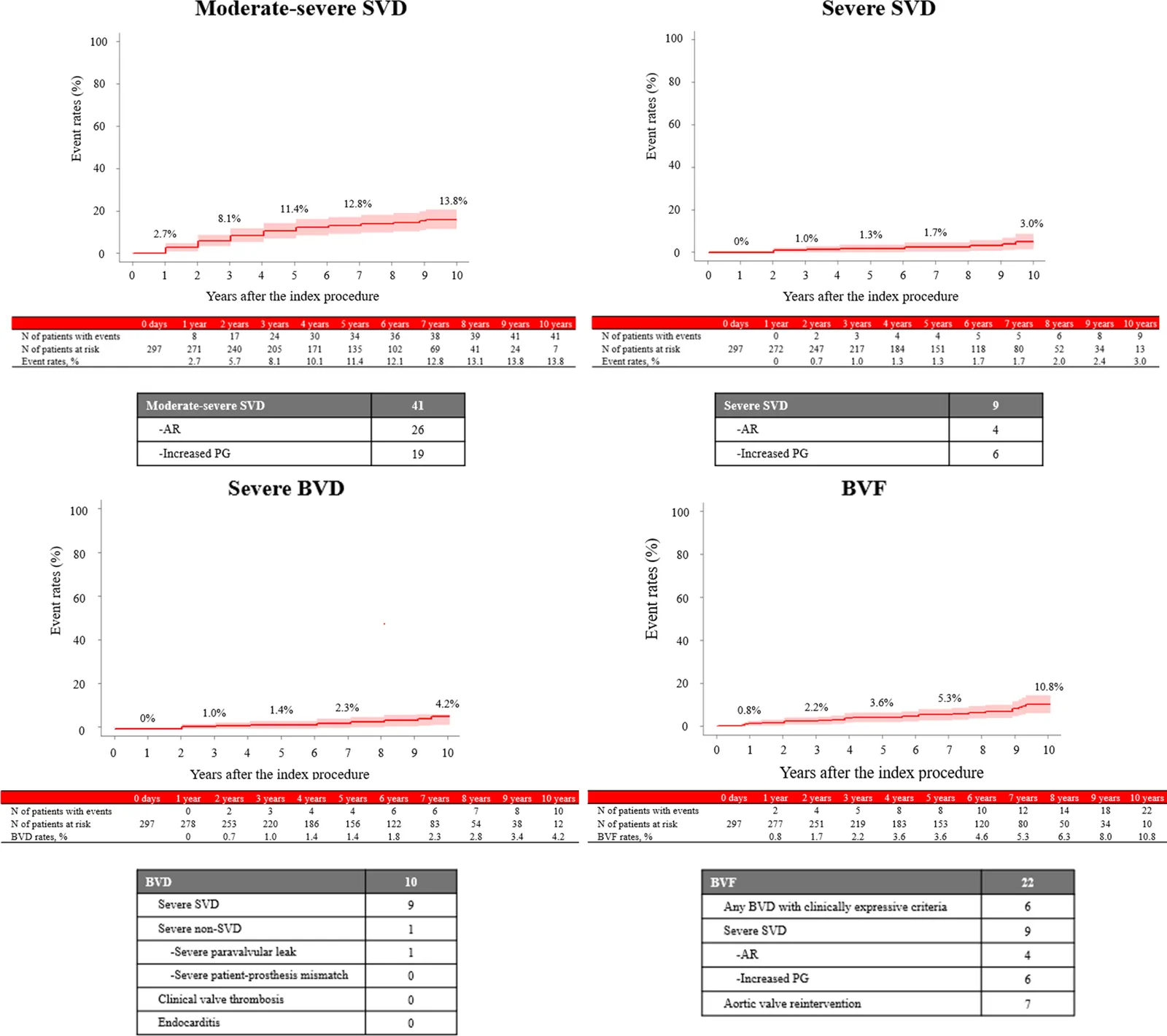
Ten-Year Cumulative Incidence of SVD, BVD, and BVF Cumulative incidence curves using the Fine–Gray competing risk model showing the incidence of structural valve deterioration (SVD), bioprosthetic valve dysfunction (BVD), and bioprosthetic valve failure (BVF) over the 10-year follow-up. AR = aortic regurgitation; BVD = bioprosthetic valve dysfunction; BVF = bioprosthetic valve failure; PG = pressure gradient; SVD = structural valve deterioration.

**Figure 5:**
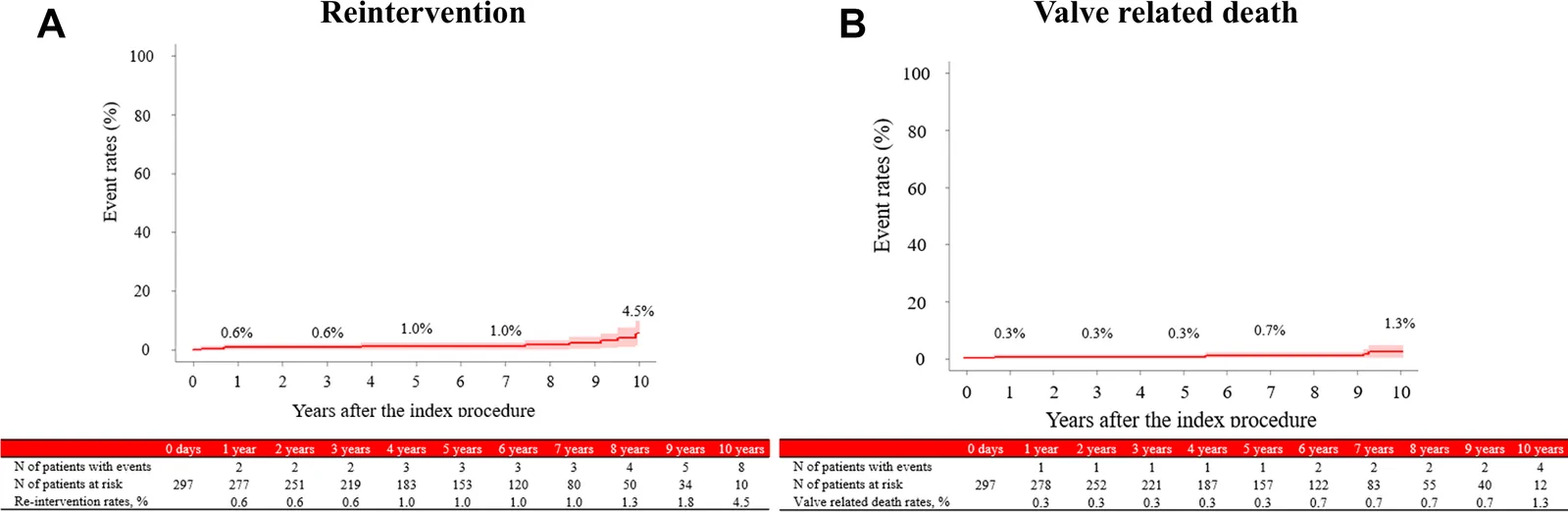
Ten-Year Cumulative Incidence of Re-intervention and Valve-Related Death Cumulative incidence curves using the Fine–Gray competing risk model showing the incidence of reintervention following (A) TAVI and (B) valve-related death over the 10-year follow-up. Abbreviation as in Figure 1.

The cumulative incidences of SVD, BVD, and BVF based on the Fine–Gray competing risk model analysis, with all-cause death treated as a competing event, are shown in Figure 4 and Supplemental Figure 1. The incidences of moderate-to-severe SVD and severe SVD at 1, 5, and 10 years were 2.7% and 0%, 11.4% and 1.3%, and 13.8% and 3.0%, respectively. The incidences of severe BVD and BVF at 1, 5, and 10 years were 0% and 0.8%, 1.4% and 3.6%, and 4.2% and 10.8%, respectively (Central Illustration). The univariate variables of BVF are presented in Table 4. In the univariate variables, no predictors of BVF at 10 years after TAVI were identified. The incidences of reintervention at 1, 5, and 10 years were 0.6%, 1.0%, and 4.5%, respectively. Eight patients underwent reintervention, including 5 due to severe SVD and 3 due to infective endocarditis. The incidences of valve-related deaths at 1, 5, and 10 years were 0.3%, 0.3%, and 1.3%, respectively (Figure 5).

**Central Illustration:**
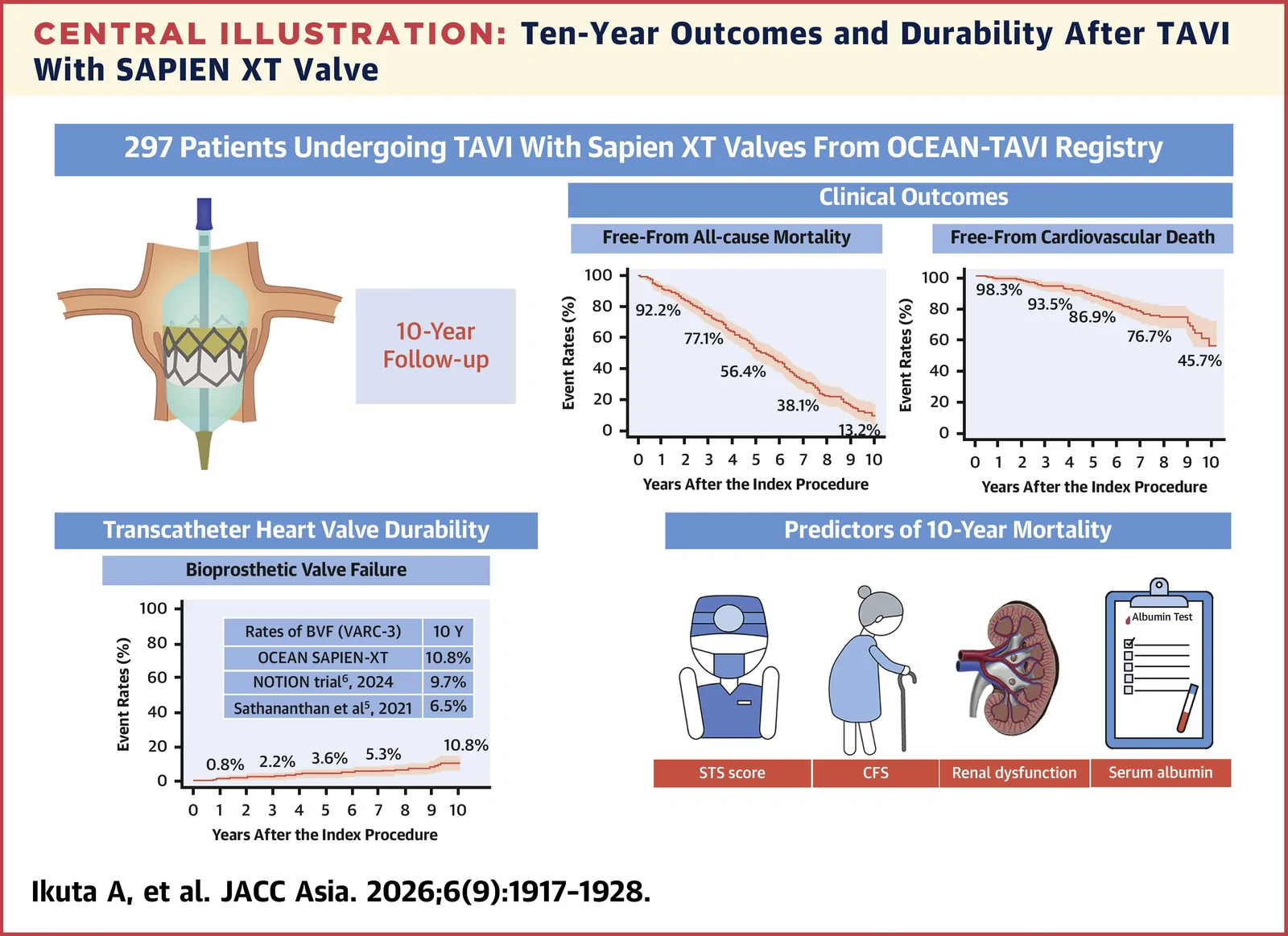
Ten-Year Outcomes and Durability After TAVI With SAPIEN XT Valve A total of 297 patients treated with transcatheter aortic valve implantation (TAVI) using the SAPIEN XT valve in the OCEAN-TAVI (Optimized Catheter Valvular Intervention–Transcatheter Aortic Valve Implantation) registry were followed for 10 years. Overall survival rates at 1, 5, and 10 years were 92.2%, 56.4%, and 13.2%, respectively, and freedom-from cardiovascular death at 1, 5, and 10 years was 93.5%, 86.9%, and 45.7%, respectively. Regarding valve durability, the cumulative incidence of bioprosthetic valve failure at 1, 5, and 10 years was 0.8%, 3.6%, and 10.8%, respectively. Predictors of 10-year mortality included Society of Thoracic Surgeons (STS) score, clinical frailty scale (CFS), renal dysfunction, and serum albumin. BVF = bioprosthetic valve failure; NOTION = The Nordic Aortic Valve Intervention Trial.

**Table 4:** Univariate Variables of BVF at 10 Years Following TAVI

	Univariate Analysis	
	HR (95% CI)	*P* Value
Age (per 1-y increase)	0.97 (0.92-1.04)	0.37
Males	1.58 (0.68-3.66)	0.29
BMI (per 1-kg/m^2^ decrease)	1.04 (0.92-1.18)	0.53
Clinical frailty scale (per 1-point increase)	1.06 (0.73-1.49)	0.77
STS risk score (per 1-point increase)	0.94 (0.82-1.06)	0.35
NYHA functional class III/IV	1.15 (0.50-2.63)	0.74
Hypertension	1.90 (0.56-6.41)	0.27
Diabetes mellitus	1.76 (0.77-4.05)	0.19
Previous myocardial infarction	1.17 (0.27-5.00)	0.84
Peripheral artery disease	1.13 (0.34-3.82)	0.84
Atrial fibrillation	0.95 (0.33-2.80)	0.93
Hemoglobin (per 1-g/dL decrease)	0.94 (0.71-1.24)	0.66
eGFR (per 1-mL/min/1.73 m^2^ decrease)	0.99 (0.97-1.02)	0.62
Serum albumin (per 1-g/dL decrease)	1.44 (0.88-2.10)	0.14
B-type natriuretic peptide (per 500-pg/mL increase)	0.91 (0.44-1.53)	0.77
Left ventricular ejection fraction (per 1.0% decrease)	0.99 (0.98-1.02)	0.83

Values are mean ± SD or n (%), unless otherwise specified.

BVF = bioprosthetic valve failure; other abbreviations as in Tables 1 and 3.

**Figure 6:**
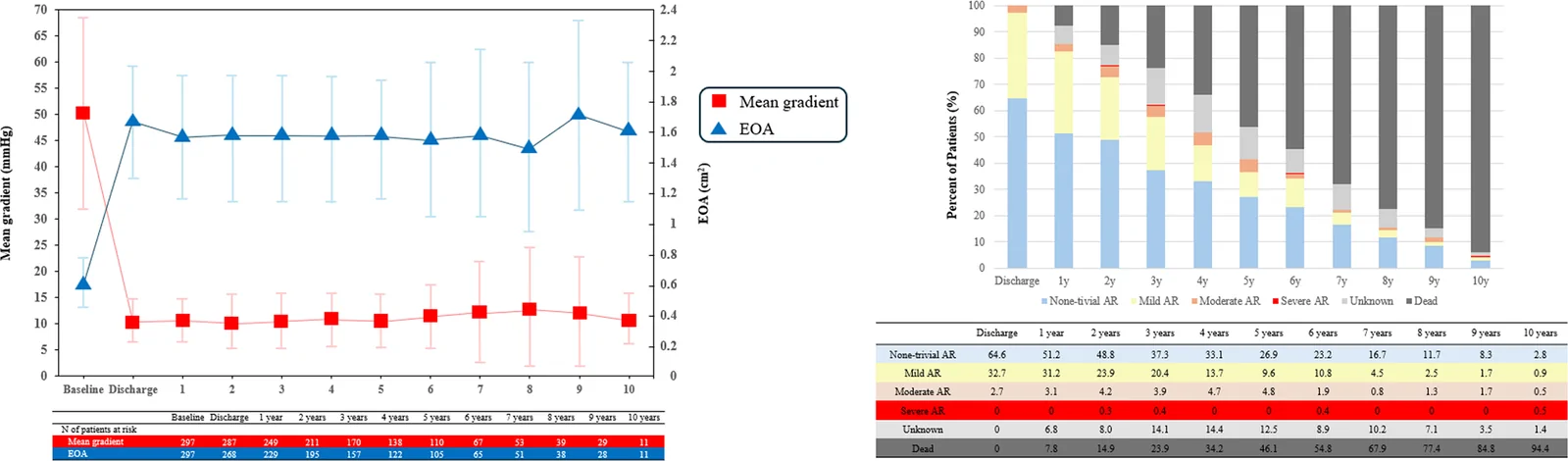
Aortic Valve Hemodynamics Over the 10-Year Follow-Up The left panel shows temporal changes in the mean transvalvular gradient and effective orifice area (EOA) over the 10-year follow-up. The right panel shows temporal changes in total AR over the same period. Abbreviations as in Figures 1 and 4.

Of the patients who achieved 10-year survival, the mPG at baseline, post-TAVI, and at 1-, 5-, and 10-year follow-up were 50.4 ± 18.2, 10.8 ± 4.2, 11.1 ± 4.2, 10.9 ± 4.9, and 10.8 ± 4.5 mm Hg, respectively. The breakdown of total AR at each follow-up is also shown in Figure 6.

### Variations in long-term mortality according to baseline characteristics

We conducted a subgroup analysis for long-term all-cause mortality based on patient characteristics (Supplemental Figure 1). Significant differences in long-term survival rates were noted across the 3 groups stratified by STS score (low-risk, STS score <4%; intermediate-risk, 4% ≤ STS score < 8%; high-risk, 8% ≤ STS score) (41.7% vs 9.2% vs 0%, respectively). Compared with the low-risk group, the HR for the intermediate-risk group was 1.88 (95% CI: 1.18-2.99; *P* < 0.01), whereas that for the high-risk group was 3.05 (95% CI: 1.91-4.88; *P* < 0.01). Patients with CFS ≥4 exhibited a significantly lower long-term survival rate than those with CFS ≤3 (3.7% vs 22.4%; HR: 1.51; 95% CI: 1.13-2.03; *P* < 0.01). Regarding renal function, compared with the group with eGFR ≥60 mL/min/1.73 m^2^, the HR for the group with eGFR <30 mL/min/1.73 m^2^ was 6.75 (95% CI: 0.85-53.3; *P* = 0.07). Although these differences were not statistically significant, a trend toward worse prognosis was observed in patients with lower eGFR. Regarding age, compared with the group ≥85 years of age, the HR for the group <80 years of age was 0.51 (95% CI: 0.34-0.77; *P* < 0.01).

## Discussion

This study offers valuable long-term data on TAVI using the first-generation balloon-expandable SAPIEN XT valve, with more than 10 years of follow-up. Such data are scarce, particularly in Asian populations. The principal findings are 3-fold.

First, the 10-year overall survival rate was 13.2%. Although extremely low survival rates, this reflects the advanced age and high-risk clinical profile of the population. Notably, this finding was observed in patients in Japan, who are generally recognized for their longevity, indicating that long-term mortality remains substantial even in such cohorts.

Second, BVF and valve-related events were relatively low despite the high mortality. BVF exhibited a cumulative incidence of 10.8%, valve-related deaths occurred in 1.3% of patients, and the re-intervention rate was 4.5%, suggesting that late outcomes were mainly unrelated to valve dysfunction.

Third, multivariable analysis identified high STS score, higher CFS, renal dysfunction, and low serum albumin level as independent predictors of mortality. These findings may contribute to optimizing patient selection and risk stratification in current TAVI practice.

### Incidence of clinical adverse events at 10 years post-TAVI

TAVI has undergone rapid developments and is now a safe and effective therapeutic approach for most patients. However, because the initial TAVI recipients were predominantly inoperable or high-surgical-risk patients, studies on long-term outcomes remain scarce. Previous studies have reported 5- and 10-year survival rates of 39.2% to 58.8% and 8.4% in high-risk populations, respectively.^5^^,^^8^, ^9^, ^10^ In the present study, the 5- and 10-year survival rates were 56.4% and 13.2%, respectively. Although data on 10-year survival in high-risk patients are limited, our findings suggest a long-term survival rate comparable to previously reported outcomes. These outcomes were reported in patients in Japan, a population generally recognized for their exceptional longevity and favorable health behaviors.^11^ Therefore, the substantial late mortality observed in this study suggests that advanced age and comorbid disease burden at the time of TAVI are major determinants of long-term outcomes even in populations with inherently longer life expectancies. Furthermore, it suggests the inherent limitations of TAVI therapy in patients with limited physiological reserve and life expectancy predominantly influenced by noncardiac conditions.

### THV durability

As TAVI is increasingly applied to lower-risk patient populations, the long-term durability of THVs remains a major concern. However, clinical data beyond 10 years are limited.^12^ In patients who survived long-term following TAVI, echocardiographic follow-up revealed preserved valve performance in most cases, consistent with previous studies.^5^^,^^13^ As in previous studies, no abrupt increase in valve-related events was noted beyond 5 years post-TAVI. However, our findings suggest a gradual increase in prosthesis-related events during the mid- to long-term follow-up period.^5^^,^^6^^,^^14^ These results emphasize the significance of ongoing structured surveillance of prosthetic valve functions beyond 5 years following the index procedure.

In this study, the 10-year cumulative incidence of BVF was 10.8%, comparable to earlier long-term studies.^5^^,^^6^ Despite the high mortality in this cohort, valve-related deaths occurred in only 1.3% of the patients, and the reintervention rate was as low as 4.5%. These findings suggest that, owing to high competing mortality, few patients survived long enough to develop valve dysfunction. In this context, TAVI with first-generation balloon-expandable THVs can be considered a sufficient treatment strategy over 10 years. However, this study targeted an older adult population with a high prevalence of comorbidities, and the elevated mortality rate may have limited the assessment of other valve-related events. Therefore, directly extrapolating these results to younger or lower-risk patients should be exercised with caution, and further accumulation of long-term follow-up data is warranted.

### Predictors of long-term all-cause mortality post-TAVI

Patients undergoing TAVI frequently present with multiple comorbidities, and several studies have investigated prognostic factors for clinical outcomes^15^, ^16^, ^17^; however, no previous studies have specifically investigated predictors of 10-year long-term outcomes. In this study, higher STS score, higher CFS, renal dysfunction, and low serum albumin level were identified as independent predictors of 10-year mortality. Several previous studies have shown the association between STS scores and long-term outcomes, typically approximately at 3 to 4 years following TAVI.^18^^,^^19^ Interestingly, regarding prognostic stratification by STS risk categories, differences are relatively small during the first few years; however, a considerable divergence in mortality was noted across the 3 groups at >10 years following TAVI. Similarly, comparison between patients ≤80 years of age and those ≥85 years of age revealed that the survival curves cross from the early years through long-term follow-up, reflecting the anticipated increase in mortality associated with advanced age. In contrast, factors, including high CFS, renal failure, and low albumin level, consistently contribute to increased mortality from the time of treatment to the 10-year period, consistent with previous studies.^20^, ^21^, ^22^

In this study, patients with high STS risk, CFS ≥4, eGFR ≤30 mL/min/1.73 m^2^, or ≥85 years of age showed extremely poor 10-year survival post-TAVI. These findings suggest that, in such populations, the clinical benefit of the procedure warrants careful consideration, rather than focusing on the long-term durability of THVs. Moreover, considering the emerging evidence supporting the durability of TAVI prostheses over 10 years, the discussion about planning subsequent valve interventions following TAVI in those populations may become less significant.^6^

### Study limitations

First, this was an observational study conducted in a high-risk population using early-generation balloon-expandable valves, potentially limiting the generalizability of our findings to lower-risk cohorts or to current-generation devices. Second, this study lacked an independent adjudication committee and echocardiographic core laboratory, likely introducing reporting bias and variability in outcome assessment. Third, although the registry was prospective, some follow-up data, particularly echocardiographic assessments, were obtained only in surviving patients, introducing a competing risk of mortality that may underestimate the true incidence of structural valve degeneration. Fourth, the study population consisted of elderly and high-risk patients. Repeat valve interventions may have been deferred and conservative management chosen instead; therefore, the rate of repeat valve intervention may have been underestimated. Fifth, survivor bias in echocardiographic follow-up may have led to an underestimation of valve deterioration, as patients with worse prognosis were less likely to survive long enough to undergo long-term echocardiographic evaluation. Although competing risk analyses were used to account for death, residual bias cannot be fully excluded. Lastly, the number of patients who survived to the 10-year follow-up was relatively small, limiting the statistical power for long-term comparisons and subgroup analyses. Therefore, these findings should be interpreted with caution.

## Conclusions

The durability of first-generation THVs appeared acceptable, largely because high mortality mitigated the impact of valve dysfunction on long-term outcomes. However, the effectiveness of TAVI in this high-risk population is evident, highlighting the significance of appropriate risk stratification to maximize its clinical value.

## Funding Support and Author Disclosures

The OCEAN–TAVI registry is supported by Edwards Lifesciences, Medtronic, Boston Scientific, Abbott Medical, and Daiichi-Sankyo Company. Drs Fuku, Shirai, Tada, Watanabe, Yamamoto, and Hayashida, have been clinical proctors for Edwards Lifesciences, Medtronic, and Abbott Medical. Dr Naganuma has been a clinical proctor for Edwards Lifesciences and Medtronic. Dr Asami has been a clinical proctor for Medtronic and Abbott Medical. All other authors have reported that they have no relationships relevant to the contents of this paper to disclose.
