# Supplementary material for: Ten-Year Clinical Outcomes and Valve Durability After Transcatheter Aortic Valve Implantation With Balloon-Expandable Valves
Source: JACC Asia. 2026 Mar 14;6(9):1917–28. doi: 10.1016/j.jacasi.2026.02.003 (PMC13554770; doi:10.1016/j.jacasi.2026.02.003)
Supplement: Supplementary file 1 [file mmc1.docx]

Supplemental Figure 1. Cumulative incidences over the 10-year follow-up in specified subgroups (a, STS score; b, CFS; c, eGFR; d, age)

CFS, clinical frailty scale; STS, Society of Thoracic Surgeons risk score; eGFR, estimated glomerular filtration rate
